# Lessons from Microbes: What Can We Learn about Equity from Unculturable Bacteria?

**DOI:** 10.1128/mSphere.01046-20

**Published:** 2020-10-28

**Authors:** Beronda L. Montgomery

**Affiliations:** a Department of Energy—Plant Research Laboratory, Michigan State University, East Lansing, Michigan, USA; b Department of Biochemistry & Molecular Biology, Michigan State University, East Lansing, Michigan, USA; c Department of Microbiology & Molecular Genetics, Michigan State University, East Lansing, Michigan, USA

**Keywords:** bacterial culture, equity, inclusion, microbiology

## Abstract

Many microbiologists exhibit a fascination with unculturable bacteria. This intrigue can be expressed through curiosity about nutrient needs, as well as about parameters such as optimal temperature, oxygen levels, minimum and optimal light, or other such environmental factors. Microbiologists study organisms’ genetic language, as well as their environment of origin, for clues about essential factors or organisms’ need for coculture to support growth and thriving. We can learn many lessons about equity and stewardship-based engagement from the ways that microbiologists seek to understand how to cultivate unculturable bacteria, including the importance of understanding an organism’s language and community, replicating aspects of the environment of origin, an organism’s occasional need to transform aspects of its environment to persist, and the critical needs to provide a range of culture conditions to support diverse organisms. These lessons from the bacterial world provide guidance applicable to addressing human inequity in scientific communities, and beyond.

## Abstract (precis)

Many microbiologists exhibit a fascination with unculturable bacteria. This intrigue can be expressed through curiosity about nutrient needs, as well as about parameters such as optimal temperature, oxygen levels, minimum and optimal light, or other such environmental factors. Microbiologists study organisms’ genetic language, as well as their environment of origin, for clues about essential factors or organisms’ need for coculture to support growth and thriving. We can learn many lessons about equity and stewardship-based engagement from the ways that microbiologists seek to understand how to cultivate unculturable bacteria, including the importance of understanding an organism’s language and community, replicating aspects of the environment of origin, an organism’s occasional need to transform aspects of its environment to persist, and the critical needs to provide a range of culture conditions to support diverse organisms.

## COMMENTARY

We can learn much from the biological organisms that we study, including the importance of interplay between individuals and environments. Organisms query environmental cues and tune or modify their behaviors to promote success in particular environments, generally dynamic and complex ones. Our fascination with unculturable bacteria in particular has much to teach us about stewardship-based engagement, mentoring, and leading in our pursuit of equity. What does it mean to be “unculturable” in our understanding of bacteria? In “Growing unculturable bacteria,” Stewart (1) specifies that “in this context, ‘unculturable’ indicates that current laboratory culturing techniques are unable to grow a given bacterium in the laboratory” (p. 4151). He goes on to say “Therefore, ‘unculturable’ does not mean ‘can never be cultured’ but, rather, signifies that we lack critical information on their biology, and this presents both challenges and opportunities” (1) (p. 4151). Indeed, “bacteria that can be grown in the laboratory are only a small fraction of the total diversity that exists in nature” (1) (p. 4151). Microbiologists generally recognize that “at all levels of bacterial phylogeny, uncultured clades that do not grow on standard media are playing critical roles … and impacting the surrounding organisms and environment” (1) (p. 4151). Other microbiologists have likewise recognized the practical importance of learning to cultivate difficult-to-cultivate bacterial strains, as “organisms of key importance to the community and the entire ecosystem in the environment or pathogens of plants and animals may be overlooked if they are unculturable” (2) (p. 1).

It is fascinating to observe the curiosity which some microbiologists demonstrate concerning the bacteria in nature that we are incapable of cultivating in laboratories and the commitment that they show to deciphering ways to do so. General responses from microbiologists about the existence of unculturable bacteria assure us that it is not that these organisms are incapable of growth but that we simply have not yet ascertained the right or essential conditions to allow them to be cultured successfully. Indeed, driven by curiosity and persistence, some scientists will commit decades to seeking out the specific critical factors needed to support these bacteria so that we can divine their magic and mystery and capture what they may contribute to our general knowledge base and ongoing existence (1, 2).

Of note, an “interesting approach to growing uncultured bacteria uses the bacteria themselves to determine the particular aspect of the environment that is important to their growth” (1) (p. 4154). It is increasingly common to use environmental sampling and nucleic acid sequencing to determine which organisms are present in a particular environment or context. We can also sequence entire genomes of organisms of interest to gain insights into which genes are present as a means to predict or comprehend what an organism needs to grow. This process requires scientists to take the time to tease apart the “language” of a particular organism, which is critical to helping us understand what key factors are important to its growth, as well as to appreciating the sheer beauty of understanding an organism itself. Microbiologists can be fascinated with this process without end. Indeed, the richness of an organism’s life, and the potential achieved or lost therein, lies hidden unless we understand its language.

Stewart (1) specifically notes that where progress has been made in supporting the growth of previously uncultivable bacteria, “the successes described … have, for the most part, combined traditional culturing methods (petri plates, liquid cultures) with new ways of making the medium more similar to the environment, including coculture with other environmental bacteria” (p. 4157). Insights from genome sequencing and proteome analyses can contribute to such progress.

Many bacteria live in complex communities in natural contexts, with each bacterial species or other entity contributing an important function (3). Bacteria are also impacted by viruses found in the community for which they serve as a biological host. Where viruses are able to successfully enter a bacterial cell and replicate, viruses can have long-term impacts on community function and bacterial evolution due to influencing information exchange and via natural selection for resistant microbes (4).

Appreciation of the importance of determining essential properties of the environment of origin, as well as the importance of coculturing with other specific organisms, has contributed significantly to expanding the range of bacteria whose growth and reproduction we can support (1, 2).

## WHAT SPECIFIC EQUITY LESSONS CAN WE LEARN FROM OUR FASCINATION WITH UNCULTURABLE BACTERIA?

Ultimately, why do unculturable bacteria fail to grow? Stewart (1) aptly declares that “the simple explanation for why these bacteria are not growing in the laboratory is that microbiologists are failing to replicate essential aspects of their environment. This is not for lack of trying or cleverness” (p. 4152). This might be considered an excellent framing to consider as we seek to understand academic failures to provide access and success for minoritized groups at parity to their proportion in U.S. demographics.

As microbiologists’ quest for cultivable bacteria relates to marginalized and minoritized colleagues, we often see only challenges, rather than opportunities. We also frequently fail to bring our creativity, cleverness, or innovation to attempts to shift the needle in regard to supporting scientists from diverse backgrounds. Certainly, we rarely see exchanges with marginalized and minoritized colleagues or long-standing disparities in supporting their success as a gateway to identifying opportunities to assess and transform our environments to serve them better—thereby serving all better, rather than our common responses of focusing on the perceived deficits of these individuals or their inability to fit “proven” and presumed meritocratic environments (5).

Our understanding of microbiologists’ fascination with unculturable bacteria offers key lessons that are beneficial to considering the long-standing inequities and very slow progress in the task of adequately diversifying academic environments—or, as Stewart put it in describing unculturable bacteria, “the culturing efforts of the last 2 centuries had managed to replicate permissive growth conditions for only a small subset of the total bacterial diversity” (1) (p. 4151). By extension, we have much work to do to understand that our institutions generally represent a “single medium” that supports the success of a limited range of individuals. If we genuinely want to support success broadly, we must be positively curious about and commit to learning the “language(s)” and providing for the needs of all—including the marginalized and the minoritized. Furthermore, we must draw on additional lessons from these fascinating organisms to rapidly and irreversibly move away from deficit framing.

It is generally well recognized that “faced with an unfamiliar environment devoid of essential factors, bacteria may, as a survival strategy, enter into a temporary state of low metabolic activity accompanied by the inability to proliferate … which may be mistaken for a constitutional resistance to culture” (2) (p. 2). This characteristic may well explain the pervasive mislabeling of some minoritized and marginalized individuals as “misfits” or resistant to acculturation, rather than appropriately judging whether the environments themselves chronically lack essential factors to broadly support individuals from diverse backgrounds (5, 6). If growth-centered perspectives and actions are to reverberate throughout institutions, we will need to select and reward leaders who are committed to and rewarded for promoting individual success, in its most extensive definition, rather than serving as gatekeepers who can determine who gets to grow in the “selective media” conditions currently pervasive in academic institutions (6).

We need to recognize that each of us has critical roles to play and essential contributions to make. We need to have authentic conversations about whether our curiosity extends beyond our interest in unculturable bacteria to seeking to understand why academic institutions have served only some well and have long maintained whiteness (Table 1). It is unjust that the minoritized and marginalized have lost opportunities to equitably pursue advancement and success, but beyond that, everyone in academic environments loses when success is not supported to its fullest extent. In losing the contributions of all, we lose the contributions to our understanding, and we lose the enrichment of our community.

**TABLE 1:** Selected factors that we accept about unculturable bacteria that we reject about minoritized and marginalized colleagues

What we accept about unculturable bacteria	False assumptions about minoritized individuals
Recognized as “metabolically active,” yet unable to grow in an environment due to our inability to “know” what critical factors are needed to cultivate and culture growth (1, 2)	Individuals are in an environment that has all needed factors to support success and thus “failure to thrive” is attributed to an individual deficit (5)
Culture conditions limit or allow only permissive conditions for a limited subset of bacterial species of known diversity (1, 2)	Our cultures are meritocratic, or with only unconscious biases, and we are adequately capable of supporting minoritized individuals; a lack of diversity is due to individuals from these backgrounds failing to succeed (6, 8)
We lack abilities to support the growth of many bacteria, referred to by Stewart (1) as “the uncultured majority”	Institutions, as well as the peers, mentors, and leaders therein, possess abilities to support success of individuals from diverse backgrounds and we have structures of accountability to do so, and thus failure to succeed is an “individual” problem (5, 6)
We need to study uncultivable bacteria to learn their “language” that communicates to us their requirements for successful culture (1, 2)	Individuals exhibiting deficits in growth need to learn the “institutional language of success” and learn to adapt to the established environment(s) to demonstrate growth and pursue success (5, 6, 8)
There is general widespread curiosity about and encouragement to investigate the permissive conditions and unique culture conditions needed to support bacteria that previously had been uncultivable (1)	Negative stereotypes about minoritized individuals are pervasive; individuals committed to “service” can support marginalized and minoritized individuals in assimilating to models of success through the “white gaze” (5, 6, 8, 9)
The environment of origin and other organisms growing in that environment may be absolutely critical to supporting growth of unculturable bacteria (1–3)	Individuals are often brought in as a “first or only” and only recently has the true importance of cohort-based recruitment and engagement started to be recognized, and it has yet to be broadly recognized as a critical goal or factor (9, 10)
Sometimes a bacterium must “transform” some aspect of the environment to make it possible for the organism to survive (1)	Minoritized individuals may be penalized for attempting to transform the environment rather than assimilating to the dominant culture (5, 8, 11, 12)

How can we fully support and attain the success of the minoritized and marginalized in the same way as we achieve breakthroughs with unculturable bacteria that derive from those new ways of promoting permissive culturing described by Stewart? We must recognize that “much of the progress in expanding the range of bacteria that can be grown has come from two related strategies: employing the environment itself as an aid in growing microbes and coculture with other bacteria from the same environment” (1) (p. 4152). Similar approaches in academic spaces would require innovation, much as studies with bacteria call for “unraveling the molecular mechanisms of unculturability and … identifying growth factors that promote the growth of previously unculturable organisms” (1) (p. 4151).

In any sphere of life, our environments can be improved by our use of stewardship-based transduction, or mentoring and leadership practices, that can be informed and inspired by what we know and love about the range of biological organisms that we study, or seek to study (5, 7). In scientific or academic contexts, we can endeavor to increase our awareness about positives, negatives, or gaps present in an environment. We can guide individuals to better perceive whether the critical factors they need for support and growth are present and available. We must have real commitment and accountability for assisting them when these critical factors are absent. We must also intentionally promote appreciation of the diverse functions that each member of our academic communities provides. Such efforts will require environmental stewards, mentors, or leaders who can appropriately help individuals build and cultivate developmental support networks that position them to achieve successful outcomes, as well as those who recognize and reward unique contributions.

Approaches that would enable us to ask questions about how individuals, especially mentors and leaders, demonstrate stewardship in science environments, rather than solely focusing on whether the individuals whom they are supporting are able to fit in the presumed-infallible environments, are critical. Lessons that we can learn from organismal assessment and microbiologists’ successes with cultivating the previously uncultivable can yield new growth-centered modes of engagement with our peers, mentors, or leaders (5–7). These stewardship-based efforts may finally lead to the transformation in equitable practice that we claim to seek and, more importantly, that we desperately need.
